# Personalia

**Published:** 1913-11-01

**Authors:** 


### Personalia.

Mr. A. Hinton Stewart has joined the board of management of the Manchester Children's Hospital, Pendlebury.

Miss Pauline Chase has consented to appeal for a "Peter Pan" cot at Charing Cross Hospital. For this purpose she will organise a series of collections during her next season at the Duke of York's Theatre, the amount required being £500. The proposal has the sympathetic support of Sir J. M. Barrie.

Much sympathy is being felt with Dr. C. Thackray Parsons, the well-known medical superintendent of Fulham Infirmary, in the loss of his wife, whose death occurred, after a brief illness, on Saturday last. Our readers, who have often had the benefit of reading Dr. Thackray Parsons' signed articles in The Hospital, and the Poor-Law world of London will share in the sorrow that has come so unexpectedly upon him, and respectfully offer their condolences, in which we sincerely join.

